# Synchronous double cancers of primary hepatocellular carcinoma and cholangiolocellular carcinoma: a case report

**DOI:** 10.1186/s40792-016-0262-2

**Published:** 2016-11-22

**Authors:** Kazuhiro Suzumura, Yasukane Asano, Tadamichi Hirano, Toshihiro Okada, Naoki Uyama, Nobuhiro Aizawa, Hiroko Iijima, Keiji Nakasho, Shuhei Nishiguchi, Jiro Fujimoto

**Affiliations:** 1 Department of Surgery, Hyogo College of Medicine, 1-1, Mukogawa, Nishinomiya, Hyogo 663-8501 Japan; 2 Department of Internal Medicine, Hyogo College of Medicine, 1-1, Mukogawa, Nishinomiya, Hyogo 663-8501 Japan; 3 Department of Pathology, Hyogo College of Medicine, 1-1, Mukogawa, Nishinomiya, Hyogo 663-8501 Japan

**Keywords:** Cholangiolocellular carcinoma, Hepatocellular carcinoma, Double cancer, Hepatectomy, Recurrence

## Abstract

Synchronous double cancers consisting of hepatocellular carcinoma (HCC) and cholangiolocellular carcinoma (CoCC) are extremely rare. We herein report a surgical case of synchronous double cancers in a patient with primary HCC and CoCC. A 45-year-old man with hepatitis B was admitted to our hospital with hepatic tumors. The level of protein induced by vitamin K antagonist (PIVKA-II) was found to be elevated. Computed tomography (CT) revealed a 23-mm tumor with early-phase enhancement and late-phase washout in the 6th segment of the liver, and a 10-mm tumor with slight early-phase enhancement and late-phase washout in the 7th segment of the liver. Magnetic resonance imaging (MRI) revealed that the two tumors in the 6th and 7th segments showed low intensity on T1-weighted images and high intensity on T2-weighted images. Based on those preoperative examinations, the liver tumors were diagnosed as multiple primary hepatocellular carcinomas. The patient underwent a posterior segmentectomy. A histopathological examination revealed that the tumor of the 6th segment of the liver was moderately differentiated HCC, and that the tumor of the 7th segment of the liver was CoCC. The postoperative course was uneventful. However, lymph node recurrence was observed 6 months later and the patient died 20 months after surgery. There are only six reported surgical cases of synchronous double primary liver cancers consisting of HCC and CoCC. We are of the opinion that curative resection may be an effective treatment for double cancer consisting of HCC and CoCC, and that it may provide long-term survival.

## Background

The incidence of synchronous double cancers consisting of different primary hepatic tumors is very low. Most cases involve patients with double cancers consisting of hepatocellular carcinoma (HCC) and intrahepatic cholangiocellular carcinoma (CCC). Cholangiolocellular carcinoma (CoCC) is a rare form of primary liver cancer that was first proposed by Steiner and Higginson in 1959 [1]. There are only five other reported cases of synchronous double primary liver cancers consisting of HCC and CoCC [2–6]. We herein report a surgical case of synchronous double cancers in a patient with primary HCC and CoCC and review the literature.

## Case presentation

A 45-year-old man with hepatitis B was admitted to our hospital because of hepatic tumors. He was administered a daily dose of 0.5 mg entecavir. He had a history of treatment for esophageal varices. The results of a physical examination were normal. A laboratory examination produced the following results: white blood cell count, 3000/μl; red blood cell count, 473 × 10^4^/μl; hemoglobin, 13.1 g/dl; platelet count, 8.7 × 10^4^/μl; albumin, 4.5 g/dl; total bilirubin, 1.7 mg/dl; and prothrombin time (percent), 79%. The alanine aminotransferase, aspartate aminotransferase, alkaline phosphatase, lactate dehydrogenase, and γ-glutamyl transpeptidase levels were within the normal ranges. The indocyanine green retention value at 15 min was 34%. The patient was hepatitis B virus-related antigen (HBsAg)-positive and HBs antibody (HBsAb)- and hepatitis C virus antibody (HCVAb)-negative. The serum HBV-DNA was not detected. The level of protein induced by vitamin K antagonist (PIVKA-II) was found to be elevated (317 mAU/ml), but the level of alpha-fetoprotein (AFP) was within the normal limits.

**Fig. 1:**
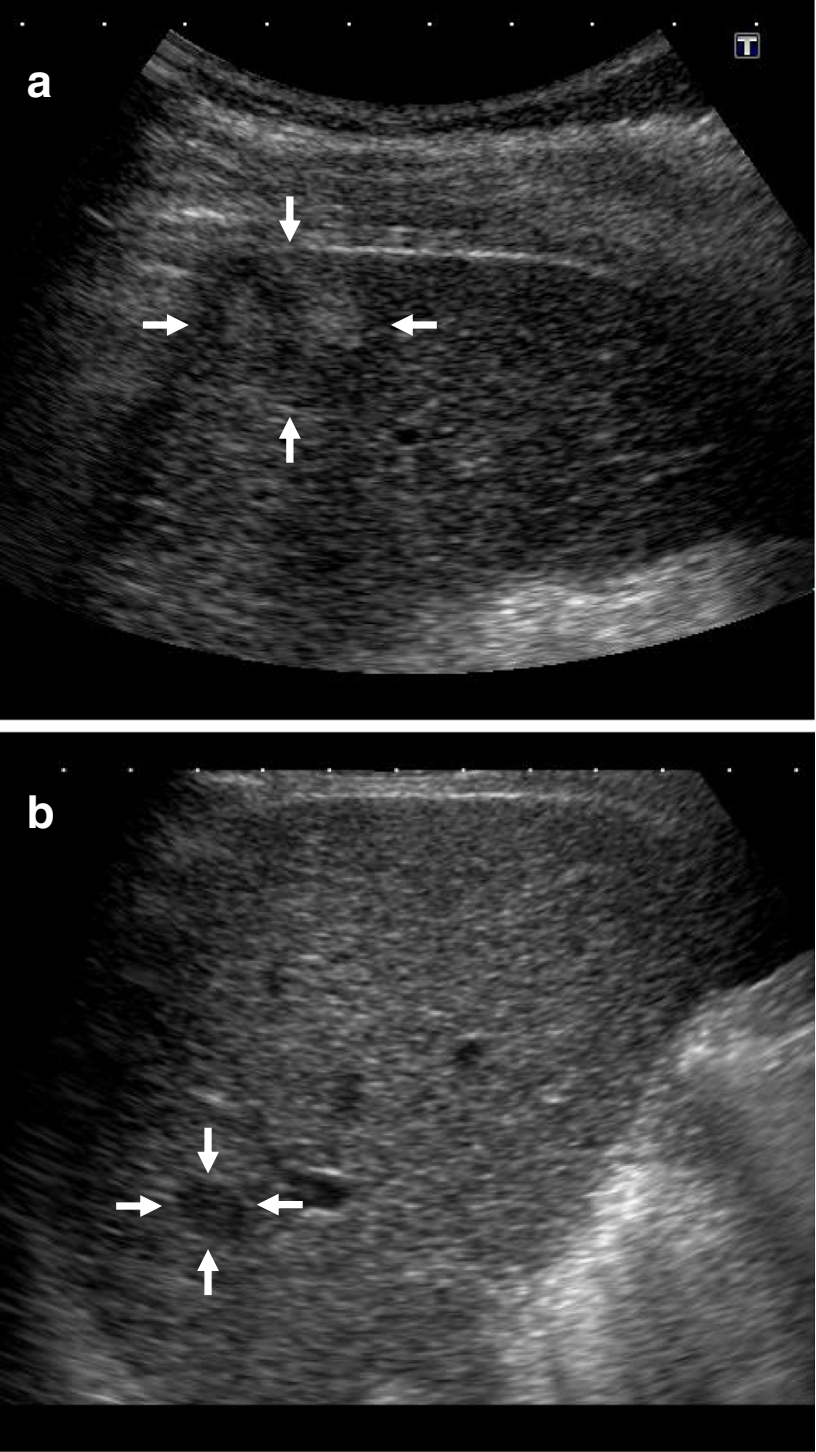
Abdominal ultrasonography (US) showed the two tumors. **a** A well-defined 23-mm hypoechoic mass that was heterogeneous on the inside was observed in the 6^th^ segment of the liver (*arrows*). **b** A slightly ill-defined 10-mm hypoechoic mass that was homogeneous on the inside was observed in the 7^th^ segment of the liver (*arrows*)

**Fig. 2:**
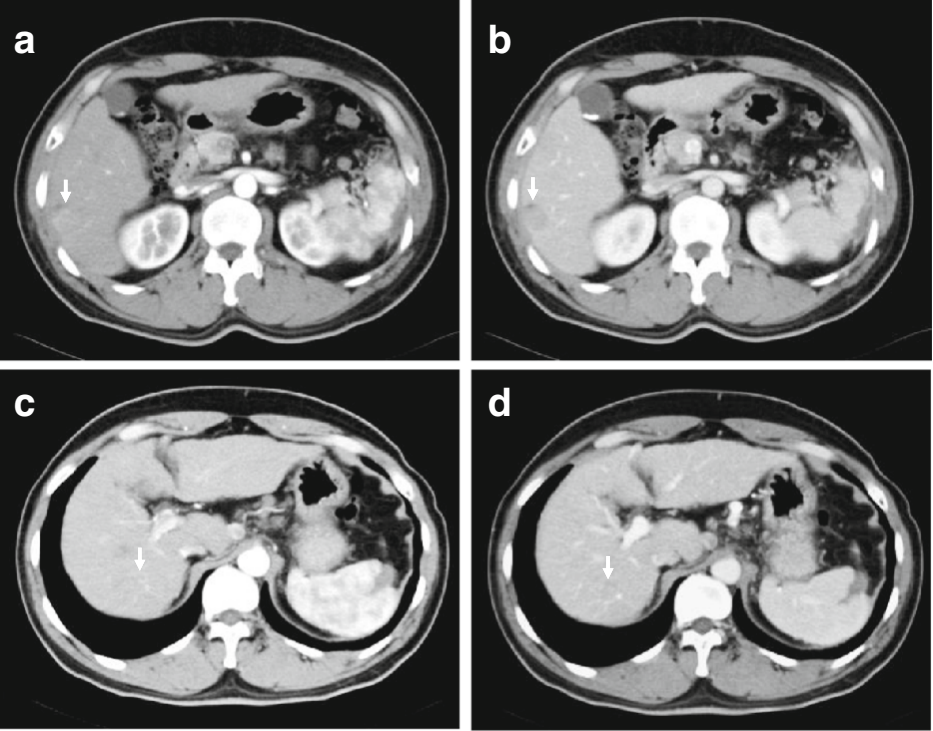
Abdominal computed tomography (CT) of the two tumors. **a** A 23-mm tumor had early-phase enhancement in the 6^th^ segment of the liver (*arrow*). **b** The tumor showed late-phase washout in the 6^th^ segment of the liver (*arrow*). **c** A 10-mm tumor showed slight early-phase enhancement in the 7^th^ segment of the liver (*arrow*). **d** The tumor showed late-phase washout in the 7^th^ segment of the liver (*arrow*)

**Fig. 3:**
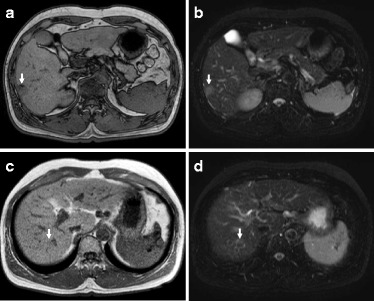
Abdominal magnetic resonance imaging (MRI) of the two tumors. **a** The tumor in the 6^th^ segment of the liver showed low intensity on T1-weighted images (*arrow*). **b** The tumor in the 6^th^ segment of the liver showed high intensity on T2-weighted images (*arrow*). **c** The tumor in the 7^th^ segment of the liver showed low intensity on T1-weighted images (*arrow*). **d** The tumor in the 7^th^ segment of the liver showed high intensity on T2-weighted images (*arrow*)

**Fig. 4:**
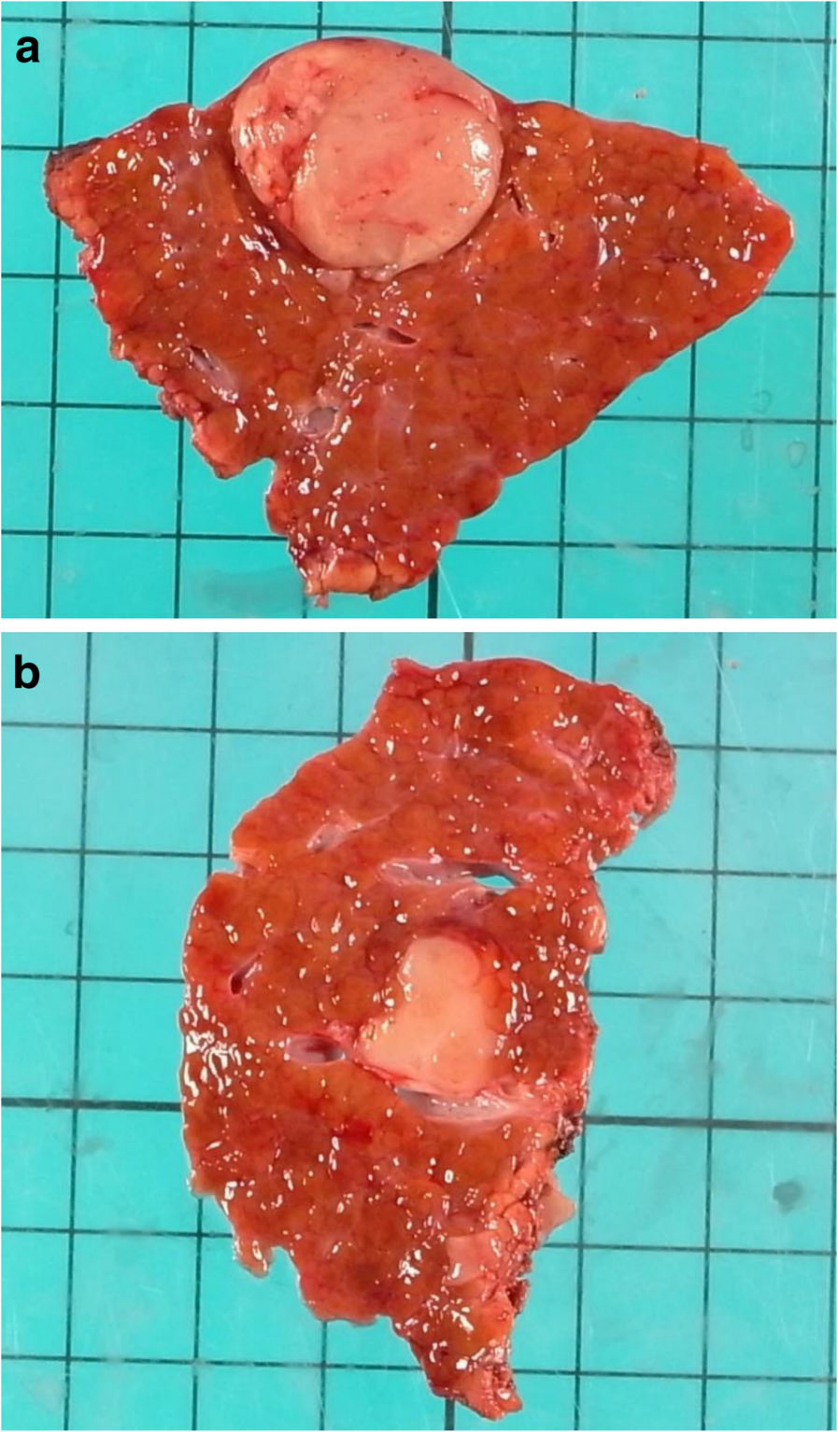
The resected specimen showed two tumors. **a** The tumor of the 6^th^ segment of the liver was a 23-mm well-defined yellowish-white soft elastic lesion. **b** The tumor of the 7^th^ segment of the liver was a 10-mm irregular yellowish-white slightly hard elastic lesion

**Fig. 5:**
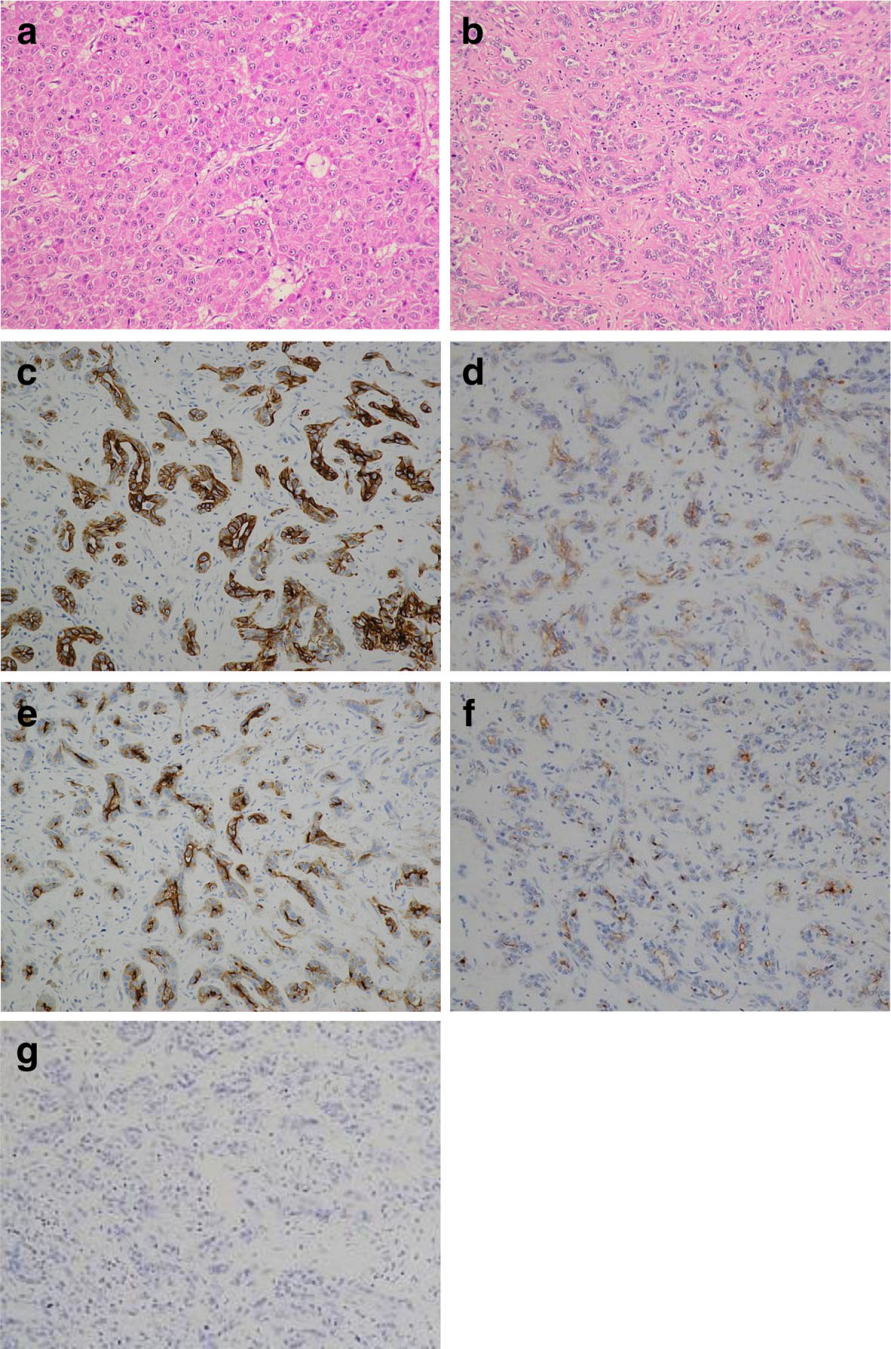
The histopathological examination of the two tumors. **a** The tumor of the 6^th^ segment of the liver was moderately differentiated hepatocellular carcinoma (Hematoxylin and eosin staining, ×200). **b** The tumor of the 7^th^ segment of the liver was cholangiolocellular carcinoma (Hematoxylin and eosin staining, ×200). **c** The immunohistochemical examination of tumor cells from the 7^th^ segment of the liver revealed that they were CK7 positive (×200). **d** The immunohistochemical examination of tumor cells from the 7^th^ segment of the liver revealed that they were CK19 positive (×200). **e** The immunohistochemical examination of tumor cells from the 7^th^ segment of the liver revealed that they were EMA positive (×200). **f** The immunohistochemical examination of tumor cells from the 7^th^ segment of the liver revealed that they were MUC1/DF3 positive (×200). **g** The immunohistochemical examination of tumor cells from the 7^th^ segment of the liver revealed that they were EpCAM negative (×200)

Abdominal ultrasonography (US) showed a well-defined 23-mm hypoechoic mass that was heterogeneous on the inside in the 6th segment of the liver (Fig. 1a), and a slightly ill-defined 10-mm hypoechoic mass that was homogeneous on the inside in the 7th segment of the liver (Fig. 1b). Abdominal computed tomography (CT) revealed a 23-mm tumor with early phase-enhancement and late-phase washout in the 6th segment of the liver (Fig. 2a, b), and a 10-mm tumor with slight early-phase enhancement and late-phase washout in the 7th segment of the liver (Fig. 2c, d). Abdominal magnetic resonance imaging (MRI) revealed that the two tumors in the 6th (Fig. 3a, b) and 7th (Fig. 3c, d) segments showed low intensity on T1-weighted images and high intensity on T2-weighted images. Based on these preoperative examinations, the liver tumors were diagnosed as multiple primary hepatocellular carcinomas. The patient underwent a posterior segmentectomy. We did not perform lymph node dissection, because we diagnosed multiple primary hepatocellular carcinomas preoperatively. The resected specimen showed that the tumor of the 6th segment of the liver was a 23-mm well-defined yellowish-white soft elastic lesion (Fig. 4a), and that the tumor of the 7th segment was a 10-mm irregular yellowish-white slightly hard elastic lesion (Fig. 4b). A histopathological examination revealed that the tumor of the 6th segment of the liver was moderately differentiated hepatocellular carcinoma (Fig. 5a), while that of the 7th segment was cholangiolocellular carcinoma (Fig. 5b). The pathological findings of the non-cancerous tissue were liver cirrhosis. An immunohistochemical examination of the tumor cells in the 7th segment of the liver was positive for CK7, CK19, EMA, and MUC1/DF3 and was negative for EpCAM (Fig. 5c–g).

The postoperative course was uneventful and the patient was discharged on postoperative day 25. However, 6 months later, lymph node recurrence of the hepatoduodenal ligament was observed. The patient died of multiple organ failure due to cancer recurrence 20 months after surgery.

### Discussion

HCC and CCC are the two main primary liver cancers. CoCC is a rare form of primary liver cancer. In 1959, Steiner and Higginson described the distinct pathological characteristic of CoCC, which is derived from the cholangioles or canals of Hering [1]. The tumor was classified as a special type of CCC [7, 8]. However, as a result of recent advances in the study and knowledge of hepatic progenitor or stem cells, CoCC is considered to originate from hepatic progenitor or stem cells [9–12].

The occurrence of synchronous double cancers consisting of different primary hepatic tumors is very low. Combined HCC and CCC is a rare primary liver tumor, and the reported incidence of combined HCC and CCC among pathologically diagnosed primary liver cancers was only 0.54% [13]. Allen and Lisa defined three types of HCC-CCC: (a) HCC and CCC are present at different sites within the same liver (double cancer type), (b) HCC and CCC are present at adjacent sites and mingle with each other but are still recognizable as distinct tumors (combined type), and (c) HCC and CCC components combined within the same tumor and indistinguishable as separate entities (mixed type) [14]. In the present case, we diagnosed a double cancer consisting of HCC and CoCC because the tumors were present at different sites within the same liver.

There are only six reports of double cancer consisting of HCC and CoCC, including our case (Table 1) [2–6]. The patients in these cases were from 45 to 71 years of age (average 65 years); five of the patients were male and one was female. Two of the six patients were HCVAb-positive, two were HBsAg-positive, and two were HBsAb-positive. In all of the six cases, the AFP or PIVKA-II levels were found to be elevated, but the level of carcinoembryonic antigen (CEA) or carbohydrate antigen 19-9 (CA19-9) was not. The average sizes of the CoCC and HCC tumors (in greatest dimension) were 1.6 cm (range 0.8–2.2) and 2.8 cm (range 1.2–4.5), respectively. In the histopathological examinations of the non-cancerous portion of the liver, three patients had chronic hepatitis and two patients had liver cirrhosis.

**Table 1:** The surgical cases of synchronous double cancers consisting of primary hepatocellular carcinoma and cholangiolocellular carcinoma

Author	Year	Age	Sex	HBsAg/HBsAb/HCVAb	AFP(ng/ml)/PIVKAII(mAU/ml)	CEA(ng/ml)/CA19-9(U/ml)	CoCC location/size	HCC location/size	Non-cancerous portion	Prognosis
Matsuda [2]	2006	70	M	(−)/ND/(+)	NR/2155	NR/NR	S7/2.2 cm	S4/4 cm	chronic hepatitis	30 months alive without recurrence
Ikeda [3]	2010	64	M	(+)/(−)/(−)	39.7/202	ND/ND	S8/2.2 cm	S8/1.7 cm	liver cirrhosis	8 months alive without recurrence
Kawano [4]	2012	68	F	(−)/(+)/(−)	30.1/105	2.9/25.1	S3/2 cm	S4/1.2 cm	ND	41 months alive without recurrence
Sunahara [5]	2013	71	M	(−)/(−)/(+)	NR/24458	NR/NR	S6/0.8 cm	S4/2.8 cm	chronic hepatitis	8 months alive without recurrence
Takata [6]	2014	71	M	(−)/(+)/(−)	333.5/140	0.6/ND	S3/1.5 cm	S7/4.5 cm	chronic hepatitis	13 months alive without recurrence
Our case	2016	45	M	(+)/ND/(−)	2.7/317	ND/ND	S7/1 cm	S6/2.3 cm	liver cirrhosis	20 months died

*ND* not described, *NR* normal range

The preoperative diagnosis of CoCC is extremely difficult using US, CT, and MRI. CoCC has been misdiagnosed as HCC, CCC, and metastatic liver cancer. Recently, some reports have examined the imaging findings in cases of CoCC [15, 16]. US shows a hypoechoic mass, CT shows uniform and complete enhancement or peripheral ring-like enhancement, and MRI shows isointensity or low intensity on T1-weighted images and high intensity on T2-weighted images. In our case, US showed a hypoechoic mass, CT showed slight early-phase enhancement and late-phase washout, and MRI showed low intensity on T1-weighted images and high intensity on T2-weighted images. However, we misdiagnosed CoCC as HCC.

A previous report indicated that the most frequent site of recurrence in surgically resected CoCC is the liver, and that no lymph node recurrence was seen [17]. Our case was the only case to show lymph node recurrence among the six reported cases of double cancer consisting of HCC and CoCC.

Ariizumi et al. [17] reported that the 5-year survival rate for patients who underwent curative surgery was significantly higher in the CoCC (75%) than in the intrahepatic CCC (33%). Among the six reported patients who underwent curative resection for double cancer consisting of HCC and CoCC, five patients remained alive without recurrence at the time of the publication of the report; the only exception was our patient. Therefore, curative resection may be an effective treatment for double cancer consisting of HCC and CoCC and may provide long-term survival.

## Conclusions

We reported a case of double primary HCC and CoCC. Synchronous double cancers consisting of HCC and CoCC are extremely rare, with only six reported cases of resected double cancer consisting of HCC and CoCC in the literature, including our own. We are of the opinion that curative resection may be an effective treatment for double cancer consisting of HCC and CoCC, and that it may provide long-term survival.
